# How to cope with emerging viral diseases: lessons from South Korea's strategy for COVID-19, and collateral damage to cardiometabolic health

**DOI:** 10.1016/j.lanwpc.2022.100581

**Published:** 2022-09-05

**Authors:** Soo Lim, Minji Sohn

**Affiliations:** Department of Internal Medicine, Seoul National University College of Medicine, Seoul National University Bundang Hospital, Seongnam, South Korea

**Keywords:** COVID-19, Quarantine, Preventive measures, Mortality, Cardiometabolic health, Public health, South Korea

## Abstract

South Korea is a unique country in many aspects in terms of its strategy against the COVID-19 pandemic. From February 2020, the South Korean government adopted active epidemiological investigations, strict isolation of affected patients, and extensive public lockdowns, which were helpful in controlling spread until the end of 2021. This stable situation in South Korea has changed dramatically since the Omicron variant—reportedly less severe but more infective than the original strain—became dominant from January 2022. From mid-February to mid-April 2022, daily cases of COVID-19 in South Korea increased steeply, reaching > 600,000 cases/day: the highest incidence rate in the world at that time. Despite this rapid increase, the South Korean government has eased its preventive strategies progressively, based on the belief in the efficacy of >80% of vaccine coverage in the population. Now, in June 2022, the COVID-19 situation in South Korea is improving. The mortality rate is 0·13%: the lowest among the 30 countries with the highest case counts. High vaccine coverage rate (87·7%), the efficient healthcare system, and active co-operation between private sectors and the central government seem to have contributed to this. However, it should also be noted that the COVID-19 pandemic and its preventive measures have had a negative influence on cardiometabolic profiles in the country. Considering the likelihood of another novel variant of SARS-CoV-2 or new infectious disease emerging in the future, understanding the situation in South Korea and the strategies flexibly adopted by its government could be beneficial for many countries.

## Introduction

The emergence of the SARS-CoV-2 virus has been posing the greatest challenge to human health worldwide since the 1918 influenza pandemic (the so-called “Spanish flu”) in the early 20^th^ century. Since the first case of coronavirus disease (COVID-19) was reported on 9 December 2019, as of 11 July 2022, 518,578,240 cases and 6,280,521 deaths had been confirmed globally.^1^ Of note, among them, 24,583 deaths had been confirmed with a ranking for case-fatality ratio 183 out of 199. SARS-CoV-2 infection induces mild symptoms in the early stage but carries the risk of progression to severe illness, including acute respiratory distress syndrome, multiorgan failure, and ultimately to death.^2^ People with diabetes mellitus, obesity, and/or cardiovascular diseases are more vulnerable to SARS-CoV-2 infection and have high mortality.^3^, ^4^, ^5^

**Figure 1:**
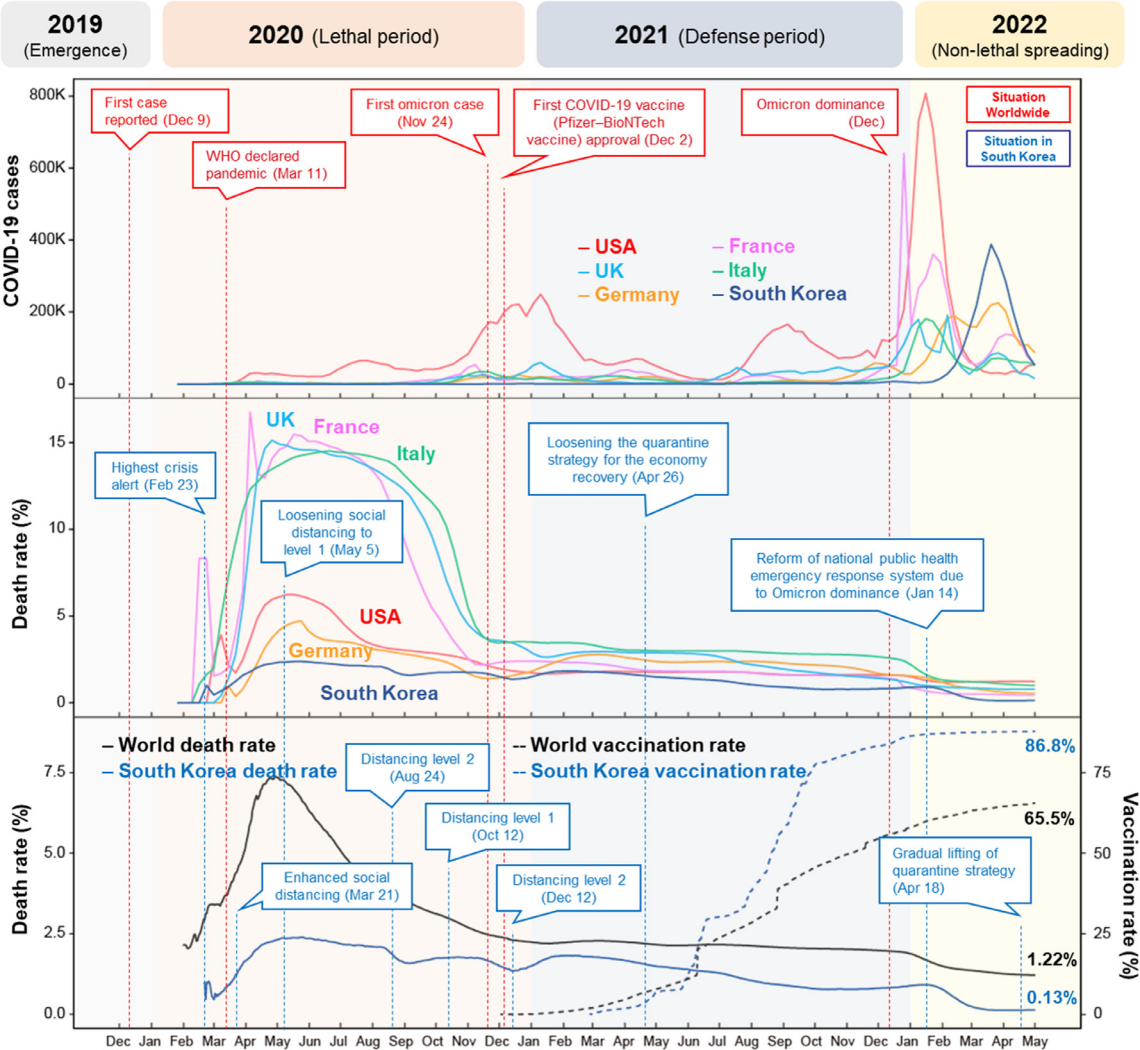
**COVID-19 cases, death, and vaccination rates in the USA, UK, Germany, France, Italy, and South Korea.** (a) COVID-19 confirmed cases; (b) Death rates; (c) vaccination rates (≥ 1) in South Korea and worldwide, with death rates. The blue arrowed text boxes show the timeline in South Korea during the COVID-19 pandemic. The red arrowed text boxes show the timeline of representative events outside South Korea at the same time. Data were retrieved from https://coronavirus.jhu.edu/map.html and https://ourworldindata.org/covid-vaccinations on May 10, 2022. Detailed information on the preventive measures adopted in South Korea is available at http://ncov.mohw.go.kr/en.

All countries have been implementing strategies against COVID-19 outbreaks. However, such preventive measures have negatively affected lifestyle patterns resulting in collateral damage to cardiometabolic and mental health. It has been difficult to adopt effective public policies because social and political situations are diverse and medical systems differ between countries. Moreover, the appearance of new variants of the SARS-CoV-2 virus, including the recent Delta^6^ and Omicron^7^ strains, has been making the situation worse.^7^ Among these, the Omicron variant, which has shown high infectivity, made it more difficult to keep the ongoing COVID-19 outbreaks under control, even with the timely development and allocation of vaccines in many countries.^8^ In addition, it is almost impossible to detect asymptomatic or mild cases of COVID-19, making quarantine measures ineffective.^9^ Thus, these mostly uncontrollable or not easily changeable factors have had profound effects on dealing with new COVID-19 cases and their severity in most countries. This has led to unique patterns in the incidence of COVID-19 and its mortality: thus, the death rate was high in early 2020 in many European countries such as the UK, France, and Italy, but it stayed low in South Korea at that time. In 2022, Omicron variants developed in the USA, France, and Germany in January to February 2022, and then spread to South Korea and increased explosively in March to April 2022, but the mortality rate remained low. Figure 1 shows these unique trends in the development of COVID-19 cases and death rates in some countries including the USA, UK, Germany, France, Italy, and South Korea.^1^^,^^10^

In this Viewpoint article, we describe how the South Korean government approached the COVID-19 pandemic by transforming the healthcare system according to the WHO Health Systems Framework.^11^ We also focus on how private sectors and the central government actively cooperated to manage the COVID-19 pandemic. In addition, we discuss the collateral damage to cardiometabolic health that developed during the COVID-19 pandemic.

## Active preventive measures in South Korea from February 2020 to January 2022

Entering the COVID-19 pandemic, the central government of South Korea transformed the country's way of supporting public health according to the WHO Health Systems Framework. Originally, 3,565 public health offices had been operated by the central government. The healthcare providers in the offices had been providing people with affordable medical care and educating the public with knowledge relevant to their needs. These government-run healthcare services were transformed into managing the SARS-CoV-2 infection immediately after the onset of the pandemic. Thus, 257 public hospitals and 479 local public healthcare centres were designated to care COVID-19 patients exclusively. Accordingly, healthcare providers were reallocated to take care of such patients. Furthermore, 619 screening posts for COVID-19 were newly built nationwide by the central government. In particular, and as of December 2021, 15,834 hospital beds were arranged for patients with moderate or severe COVID-19, aiming for an occupancy rate of 70%.

**Figure 2:**
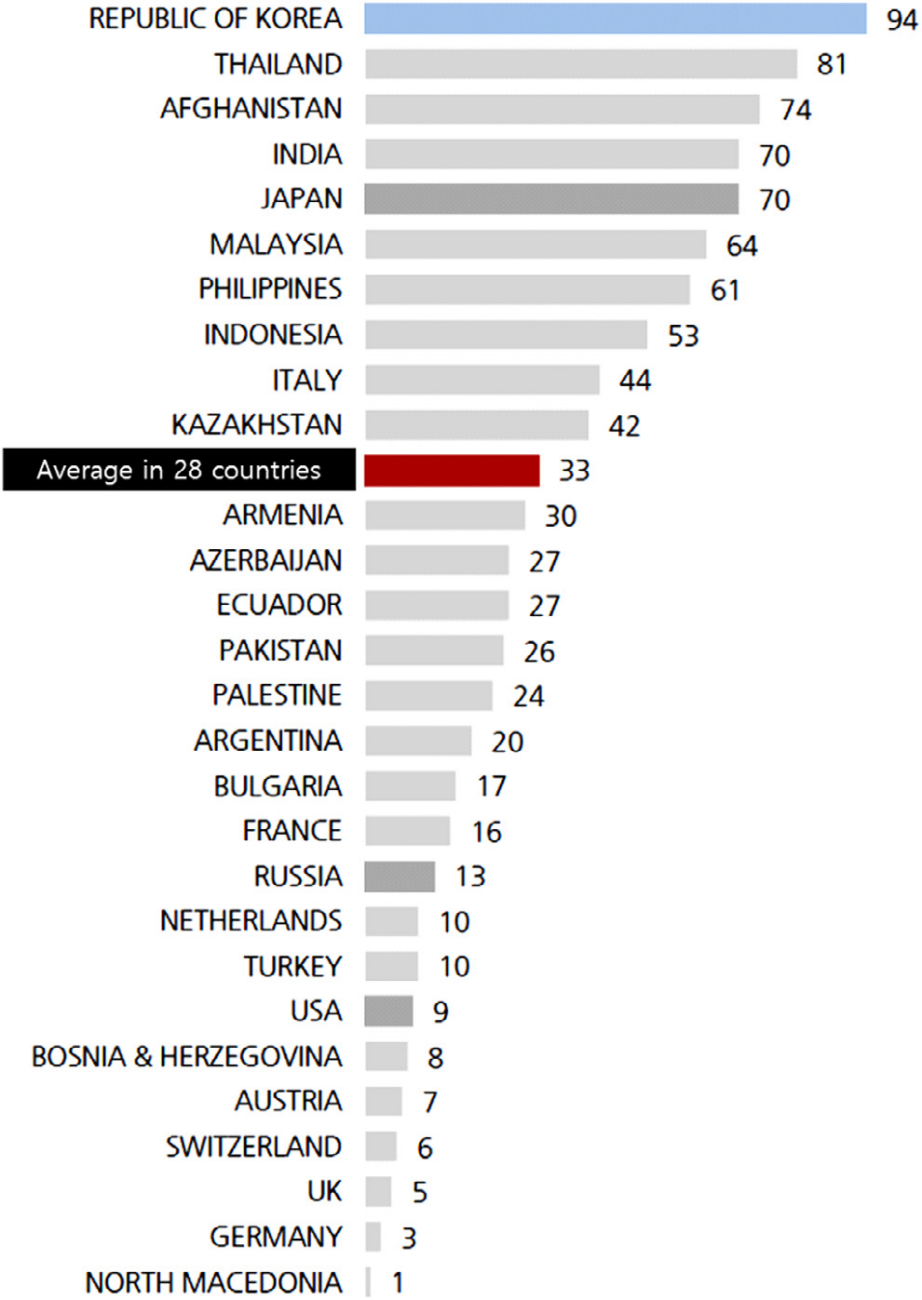
**Percentage of people wearing masks for protection against COVID-19 in 28 countries.** Data were retrieved in March 2020 (“Coronavirus: A Vast Scared Majority Around the World”; a translation from the Korean text). (https://www.gallup.co.kr/gallupdb/reportContent.asp?seqNo=1100).

From February 2020, the South Korean government adopted severe preventive measures including epidemiological investigations of the possible routes of infection, strict isolation of affected patients, and extensive public lockdowns. For individuals, there were several essential rules. Rule 1: Stay home for 3–4 days if you feel unwell. Rule 2: Keep a distance of two arms’ length from others. Rule 3: Wash your hands for at least 30 s. Rule 4: Cough or sneeze into your sleeve or elbow. Rule 5: Ventilate your dwelling at least twice a day and disinfect regularly. Moreover, high-risk groups with underlying health conditions were strongly recommended to wear N95 or KF94 masks. It was also advised to wear a mask in the following situations: taking care of persons suspected of having COVID-19 infection; upon developing respiratory symptoms; when visiting a medical facility for the elderly or disabled; or when using an indoor facility. Among these, wearing a mask was counted as one of the most effective preventive measures.^12^ Of note, from the beginning of the pandemic, wearing a mask was advised strongly, whereas in several countries, particularly in Europe,^13^ it had been discouraged by health authorities unless the individual was sick, given the lack of evidence on its efficacy^14^ and the expectation of herd immunity (the indirect protection from an infectious disease that arises when a sufficient percentage of a given population has become immune through previous infections or vaccination). In South Korea, the government imposed a fine on those who objected to wearing masks. Additionally, the mask supply was strictly managed to allow everyone to purchase a certain quantity at a reasonable price. Accordingly, in an international survey, the reported rate of wearing face masks among South Koreans was 94%, which was the highest among 28 countries (Figure 2).

These strategies taken at the central government level and the co-operation from the general public in South Korea were very effective in reducing the incidence of new cases in South Korea in 2020, while there were explosive increases in the USA and several European countries (Figure 1).

With respect to the health information systems, in the early phase of COVID-19 infections in 2020, the South Korean government built a designated website that showed COVID-19-related health information to give the public relevant information and enhance governmental transparency. Information on the website included the symptoms of SARS-CoV-2 infection; what individuals suspicious of having been infected were supposed to do; the trends in the prevalence of confirmed cases, cases in critical conditions, and deaths; vaccination coverage rate; and information about COVID-19 emergency care.

To provide essential medicines and financing, the National Health Insurance Service, operated by the central government, has covered all COVID-19-related costs—including vaccination, diagnosis, and treatment including medications—free of charge. In terms of leadership and governance, the South Korean government implemented an intensive epidemiological investigation starting from February 2020. Tracing the potential routes of infection, isolating patients with COVID-19, and imposing public lockdowns were all operated by the central government. It also conducted a campaign to prevent the spread of COVID-19, which included wearing a mask, washing hands thoroughly and frequently, and participating in social distancing (**Supplementary Table S1**). These active measures made it possible to respond to the COVID-19 pandemic faster and more effectively than other countries with privatized medical systems.^15^

Furthermore, COVID-19 tests and vaccinations were conducted in many private clinics in response to the central government's request. Healthcare providers in the private clinics actively participated by reporting suspicious cases of COVID-19 and the number of vaccinations. Medications for COVID-19 were prescribed in private clinics under supervision of the central government. Some private hospitals yielded their beds voluntarily for affected patients. With this co-operation between the private sector and the public healthcare system, people found it easy to access medical facilities for COVID-19 testing, vaccination, and treatment.

## Systematic changes in strategies against COVID-19 in South Korea from February 2022

The stable COVID-19 situation has changed dramatically since the Omicron variant—reportedly less severe but more infective than the original strain—became dominant from January 2022. Daily cases of COVID-19 (mainly the Omicron variant) increased steeply thereafter. Despite this rapid increase, the South Korean government has eased its preventive strategies progressively, allowing private gatherings, extending the business hours of restaurants and cafés, and abandoning the lockdown of public spaces. With decreased numbers of patients with moderate to severe COVID-19, as of 5 July 2022, only 10% of the operating 3,417 public COVID-19 beds were used actively. This was based on the belief that over 80% of vaccine coverage in the population in early 2022—including the elderly and people at high risk—would be helpful in reducing the number of severe cases and mortality.

It is also considered that the effectiveness of social distancing measures has been reduced because of the high transmissibility of the Omicron variant compared with the previous original or Delta variant wave. In fact, the incidence of new cases of COVID-19 increased dramatically in our country to over 600,000 per day in March 2022, and this increasing trend has continued for two months to the third week of April 2022. As a result, the total number of cases reached over 1,740,000: approximately 34% of the population of South Korea. Healthcare professionals and governmental authorities estimated that more than 60% of the population had been infected with COVID-19, based on the calculation that the total case number would be 2.3-fold that of the observed population who had been infected with COVID-19 when unidentified infected cases were considered.^16^

After the infection rate reached the “herd immunity” level (for example, 67% of a given population),^17^ from 23 April 2022, the daily rate of new cases decreased rapidly to fewer than 100,000/day. With these promising data, the South Korean government downgraded COVID-19 from first to second class in its classification of infectious diseases, meaning that a person infected with COVID-19 no longer needs to be reported immediately. From 3 May 2022, the daily rate of new cases dropped to fewer than 50,000/day. Of note, the recent rapid decline was observed during a gradual lifting of social distancing measures.

As of May 2022, some preventive measures are still in place in South Korea, even though most social distancing rules have been relaxed. Indoor mask wearing during assemblies, concerts, and social events—particularly when a 2-m distance cannot be maintained—is mandatory. Personal hygiene routines for infection prevention, such as hand washing, ventilation of dwellings, and disinfection, have become more important. The public is asked to incorporate general hygiene practices in their daily routines for the health and safety of all. Protection of high-risk groups, including the unvaccinated and the elderly, is still a high priority for monitoring. As such, the current public health measures are being strengthened for facilities or nursing homes where people gather and are at higher risk for COVID-19.

One intriguing fact about South Korea is the very low mortality from COVID-19. According to legitimate data, it is 0·13% (Table 1), which is the lowest among the 30 countries with the highest case counts. This figure is only a quarter of those reported in other countries including the USA (1·22%), Italy (0·99%), the UK (0·79%), Germany (0·55%), and France (0·51%).^1^ There are several possible explanations for this low severity in South Korea. First, there has been a very high vaccine coverage rate against COVID-19: 87·7% for the first vaccine; 86·8% for a booster dose; and 64·5% for the second booster.^10^ A recent study showed that vaccinated Koreans had a significantly lower risk of a severe course of the disease,^18^ supporting the effectiveness of the anti-COVID-19 vaccine in reducing its severity, although it was not effective in preventing contraction. Second, the effective medical healthcare system in South Korea is convenient and inexpensive for citizens to access. The major hospitals are reasonably well equipped with intensive care units (ICUs) and essential equipment for severe cases of COVID-19 such as extracorporeal membrane oxygenation systems. Third, there is proactive management for the elderly and people at high risk. These groups are prioritized to receive vaccination and to be admitted first to an ICU if necessary. Moreover, they are the first to be given oral antiviral medications. Fourth, there is active co-operation between private hospitals, public sectors, and the central government. When the central government introduced the preventive measures against COVID-19, the public followed them promptly. Immediately after the distribution of COVID-19 vaccines by the central government, the private hospitals offered them to people based on pre-adjudicated priority. In addition, the public healthcare centres received patient reports from private hospitals and incorporated them into investigative epidemiology. Once the public health centres had produced tangible results from their epidemiological investigation, they reported these to the central government and shared them with private hospitals, which again helped the government update preventive measures. Essential medical facilities have been monitored and distributed to critically ill patients first by orchestrated co-operation between the central government, local governments, and professional medical societies. Moreover, South Korea started a fourth round of vaccinations for the elderly from April 2022.

**Table 1:** Global cases and death counts in 15 countries with the highest case counts as of 12 May 2022.

	Country	Total confirmed cases	Total deaths	Fatality rate (%)	Percentage of population fully vaccinated^a^	Population
1	USA^b^	84,032,416	1,025,992	1.22	66.18%	334,607,037
2	India	43,116,600	524,181	1.22	61.74%	1,405,198,953
3	Brazil	30,639,130	664,700	2.17	76.63%	215,365,259
4	France^d^	29,097,570	147,159	0.51	77.93%	65,541,715
5	Germany^c^	25,665,910	137,628	0.54	76.77%	84,281,420
6	UK^d^	22,159,615	176,708	0.80	72.91%	68,548,221
7	Russia^b^	18,245,394	377,359	2.07	50.43%	146,050,656
8	South Korea	17,694,677	23,554	0.13	86.83%	51,351,115
9	Italy	16,954,784	164,976	0.97	79.36%	60,296,749
10	Turkey	15,050,227	98,878	0.66	62.36%	86,024,294
11	Spain^e^	12,058,888	105,123	0.87	86.46%	46,788,405
12	Vietnam	10,690,471	43,063	0.40	NA	98,964,940
13	Argentina^b^	9,101,319	128,729	1.41	81.64%	45,965,910
14	Japan	8,217,978	29,935	0.36	80.55%	125,757,594
15	Netherlands^f^	8,065,852	22,292	0.28	72.23%	17,205,274

a Data were retrieved from https://ourworldindata.org/covid-vaccinations#source-information-country-by-country on 1 May 2022.

b Vaccination data as of 30 April.

c Vaccination data as of 29 April.

d Vaccination data as of 28 April.

e Vaccination data as of 27 April. ^^Vaccination data as of 26 April.

f Vaccination data as of 23 April. A person is considered fully vaccinated if they have received a single-dose vaccine or both doses of a two-dose vaccine. NA, not available.

## Collateral damage to cardiometabolic health during the COVID-19 pandemic

From a different perspective, the COVID-19 pandemic and its preventive measures impacted negatively on metabolic and cardiovascular health in a huge number of people worldwide. Decreased physical activity and unhealthy dietary patterns linked to preventive principles such as social distancing and lockdown appear to have been the key factors mediating this.^19^ Thus, it was reported that home confinement caused by COVID-19 and quarantine protocols had unfavourable effects on physical activity and food consumption patterns.^20^^,^^21^ According to Statistics Korea data, online food shopping has increased by 66.1% from 1,292,900,000,000 Korean won (KRW) [1,047,306,601 USD] in March 2019 to 2,147,200,000,000 KRW [1,739,327,663 USD] in March 2020.^22^ Of note, the number of deliveries has increased by 9% from 31 January to 17 February compared with 3–20 January 2020, and by 11% during 1–16 February compared with 6–21 January 2020, when the virus had spread across the country.^23^

Overeating and irregular eating habits such as snack consumption between meals or late-night snacking increased significantly during the COVID-19 home confinement period.^20^ Fast-food consumption is known to be associated with weight gain, as it typically contains high levels of fat, sugar, and sodium.^24^ Thus, physical activity has decreased and unhealthy food consumption has increased during the pandemic compared with the pre-COVID-19 period in South Korea.^25^ This is likely to lead to significant increases in cardiometabolic risk factors in subjects with metabolic impairment. In fact, in the 2019–2020 season, the proportion of subjects with metabolic syndrome increased significantly by 21% compared with the 2018–2019 season.^26^ Along with elevated body mass index and systolic blood pressure, the 10-year risk for cardiovascular disease also increased compared with the previous three years.^26^ Such worsening of metabolic components has been confirmed in other countries.^27^^,^^28^

Mental health is an important issue during the COVID-19 pandemic, and is interrelated with cardiometabolic health.^29^ Hospital admissions for COVID-19 infection associated with preventive measures such as lockdowns, closing public spaces, and school shutdowns, must have affected people's psychological aspects negatively. A study reported that COVID-19 associated preventive measures were associated with deterioration in mental health, including the “CORONA blues”, which is a neologism indicating a melancholy or a lethargy one can feel when one's daily life is drastically changed by the spread of COVID-19.^30^ The COVID-19 Mental Disorders Collaborators reported that the COVID-19 global pandemic led to a >25% increase in major depressive disorders and anxiety disorders in 2020.^31^

In a different context, the preventive measures against the COVID-19 outbreak implemented in many countries are likely to contribute to vitamin D deficiency/insufficiency,^32^ which is linked to type 2 diabetes^33^ and cardiovascular disease.^34^ Substantial evidence suggests a significant association between vitamin D insufficiency/deficiency and COVID-19 susceptibility and its severity.^35^ Thus, during the COVID-19 pandemic and associated preventive procedures and thereafter, nationwide strategies to prevent deterioration in cardiometabolic health are necessary.^36^ Promotional activities on doing regular exercise and consuming healthy food are strongly recommended to mitigate the unfavourable impact of COVID-19 and related quarantine protocols on metabolic risks.^37^ Therefore, it is of utmost importance to evaluate the long-term impact on healthcare practices worldwide after the pandemic is over.

## Lessons from South Korea's strategy for COVID-19

Several issues should be discussed regarding how South Korea has dealt with this pandemic. The country maintained an active strategy for preventing the spread of COVID-19 (**Supplementary Table S2**), and this was found to be effective with a low mortality rate over 2020–2021. Timely and regular vaccination is also required to prevent or decrease severity from serious infectious diseases. Orchestrated effort among individual, private sector, and public domains is needed to manage highly contagious infectious diseases.

At present, the COVID-19 pandemic has not yet ended, and it is not yet clear whether the strategies adopted by the South Korean government were optimal. In particular, strong preventive measures such as the aggressive “trace, test, and treat” programme conducted by the central government (and later abandoned) raised concerns about privacy issues of collecting personal data about using credit cards, a history of phone calls, and records of access to public spaces.^38^ On the other side, the COVID-19 pandemic and its preventive measures had a negative influence on cardiometabolic profiles in subjects with pre-existing metabolic impairments. Thus, from a long-term perspective, encouraging regular exercise and healthy dietary habits is strongly recommended to prevent or mitigate the unfavourable impact of COVID-19 and related preventive policies on cardiometabolic risk.

As there is a chance of facing another novel viral variant or new infectious disease in the future, we must prepare the most effective measures to defeat them. From the example of South Korea, it is obvious that active surveillance and isolation and comprehensive management of infected cases are essential. Overall, rapid implementation of preventive strategies and comprehensive support for healthcare agencies will be essential for countries to deal with any highly contagious and dangerous diseases that might emerge in the future.

## Contributors

SL conceptualized the manuscript. SL and MS wrote the original draft of the manuscript. Both authors reviewed and edited the manuscript.

## Declaration of interests

We declare no competing interests.

## Appendix Supplementary materials

Image, application 1
