# Supplementary material for: How to cope with emerging viral diseases: lessons from South Korea's strategy for COVID-19, and collateral damage to cardiometabolic health
Source: Lancet Reg Health West Pac. 2022 Sep 5;30:100581. doi: 10.1016/j.lanwpc.2022.100581 (PMC9442269; doi:10.1016/j.lanwpc.2022.100581)
Supplement: Supplementary file 1 [file mmc1.docx]

| Supplementary Table S1. Social distancing system during the COVID-19 pandemic in South Korea | | | | |
| --- | --- | --- | --- | --- |
| Classification | Level 1 | Level 2 | Level 3 | Level 4 |
| Definition | Contained and stable | Local transmission / Cap on gathering size | Regional transmission / Ban on gatherings | Full-blown nationwide transmission / Ban on going out |
| Decision/Adjustment authorities | City/province, CDSCH | City/province, CDSCH | City/province, CDSCH | CDSCH |
| Criteria | < 1 case per 100,000 people (weekly average)   - Nationwide: < 500 - Greater Seoul: < 250 | ≥ 1 case per 100,000 people (weekly average > threshold for 3+ days)   - Nationwide: ≥ 500 - Greater Seoul: ≥ 250 | ≥ 2 cases per 100,000 people (weekly average > threshold for 3+ days)   - Nationwide: ≥ 1,000 - Greater Seoul: ≥ 500 | ≥ 4 cases per 100,000 people (weekly average > threshold for 3+ days)   - Nationwide: ≥ 2,000 - Greater Seoul: ≥ 1,000 |
| Private gatherings | Comply with COVID-19 protocols | Up to 8 people (gatherings of 9+ prohibited) | Up to 4 people (gatherings of 5+ prohibited) | Up to 2 people after 18:00 hrs. (gatherings of 3+ prohibited)  ^※^Private gatherings of up to 4 persons permitted until 18:00 hrs. |
|  | - Fully/partially vaccinated people not counted for immediate family gatherings - Fully vaccinated people not counted for private gatherings (except Level 4) | | | |
| Events | 500+ people only with advance reporting to local authorities | 100+ people prohibited | 50+ people prohibited | Events prohibited |
|  | - Fully vaccinated people not counted for events | | | |
| Assemblies | 500+ people prohibited | 100+ people prohibited | 50+ people prohibited | Prohibited except 1-person protests |
|  | - Fully vaccinated people not counted for assemblies | | | |
| CDSCH: The Central Disaster and Safety Countermeasure Headquarters, South Korea | | | | |

| **Supplementary Table S2. The main response measures against the COVID-19 pandemic adopted by South Korea** | |
| --- | --- |
| **Date** | **Policies in detail** |
| 23 Feb 2020 | Given the wide spread of SARS-CoV-2 virus with 100 confirmed cases and the first death, the national crisis alert was raised to the highest level of the infectious disease crisis response. |
| 29 Feb 2020 | To avoid superspreading of SARS-CoV-2 virus, social distancing was adopted. |
| 9 Mar 2020 | A policy for a five-day rotation system for purchasing masks was introduced. 48 million masks were supplied between 9 Mar and 15 Mar. |
| 21 Mar 2020 | Enhanced social distancing was practised. |
| 24 Dec 2020 | Finalized contract to attain Pfizer and Janssen vaccines: 20 million and 6 million doses, respectively. |
| 26 Apr 2021 | The quarantine strategy was loosened for economic recovery. |
| 14 Jan 2022 | The national public health emergency response system was reformed due to Omicron dominance. |
| 18 Apr 2022 | The quarantine strategy was lifted gradually. Social distancing restrictions were lifted. Instead, emphasis was placed on maintaining daily practices such as hand washing, ventilation, and disinfection. |
